# Real-time PCR–based detection of *Mycoplasma agalactiae* in sheep bulk tank milk to support flock-level epidemiology

**DOI:** 10.1007/s11259-026-11445-4

**Published:** 2026-08-06

**Authors:** Carla Cacciotto, Tania Carta, Franca Mannu, Rosanna Zobba, Emanuela Bazzoni, Ignazio Ibba, Danilo Muggianu, Francesco Turrini, Marco Pittau, Vincenzo Carcangiu, Alberto Alberti

**Affiliations:** 1 Department of Veterinary Medicine, University of Sassari, Via Vienna 2, Sassari, 07100 Italy; 2 Mediterranean Center for Disease Control, Sassari, 07100 Italy; 3 Nurex, S.r.l, Sassari, 07100 Italy; 4 LaoreSardegna, Cagliari, 09123 Italy

**Keywords:** Sheep dairy farms, Molecular diagnostics, Within-herd prevalence, Milk-based monitoring

## Abstract

This study presents the development and large-scale field application of a real-time PCR assay designed for the detection of *Mycoplasma agalactiae* in bulk tank milk from dairy sheep farms. The assay demonstrated high analytical specificity and sensitivity, enabling reliable identification of low levels of target DNA typically present in bulk milk samples. Following analytical validation, the test was used to estimate within-herd prevalence and was applied in a regional surveillance program encompassing more than 900 sheep farms across Sardinia, Italy. The findings reveal widespread but often low-prevalence circulation of *M. agalactiae* within Sardinian flocks. The real-time PCR assay proved effective for identifying infected herds through a non-invasive, cost-efficient sampling matrix, underscoring its value as a practical tool for early detection and herd-level surveillance. This approach provides a scalable framework for monitoring contagious agalactia in areas with high densities of small-ruminant farms and supports the establishment of sustainable milk-based surveillance systems.

**Supplementary Information:**

The online version contains supplementary material available at https://doi.org/10.1007/s11259-026-11445-4.

## Introduction

Contagious agalactia (CA) is a significant infectious disease of global concern affecting small ruminants and it is classified as a notifiable disease by the World Organization for Animal Health.(Agnone et al. 2013; Migliore et al. 2021) CA *sensu stricto* is caused by *Mycoplasma agalactiae* (*M. agalactiae*), a small wall-less bacterium belonging to the class *Mollicutes*. Clinically, CA is predominantly characterized by mastitis, resulting in a marked reduction in milk yield, and is frequently associated with other symptoms including keratoconjunctivitis, arthritis, and, less commonly, abortion. Due to its high morbidity (up to 100%), CA exerts a substantial economic burden on the small ruminant dairy sector, particularly in regions where this form of livestock production represents a major component of the agricultural economy, such as Sardinia.(De Azevedo et al. 2006; Migliore et al. 2021) Being a CA endemic region, Sardinia represents a critical area for the study and control of CA due to its dense population of small ruminants and the dominance of milk-oriented production systems. The island hosts approximately 3,019,108 sheep and 281,569 goats, representing 57.13% and 41.67% of the national totals, respectively. Sardinian farms contribute 68.92% of Italy’s sheep milk and 57.30% of Italian goat milk production and account for approximately 10% of total ovine milk output in Europe. The annual regional production of sheep and goat cheese is estimated at around 60,000 tons, with nearly 30,000 tons certified under the Protected Designation of Origin (PDO) label (Laore, 2019, Dati sull’allevamento ovino, caprino e bovino da latte in Sardegna, https://www.sardegnaagricoltura.it/documenti/14_43_20200904094410.pdf). CA has been introduced in Sardinia since 1980, with recurring outbreaks in the following decades, causing considerable losses in milk production, animal welfare issues, and long-term economic consequences.

The control of CA remains challenging, primarily due to the persistent nature of *M. agalactiae* within herds and its transmission through direct contact, contaminated milk, and fomites.(Loria et al. 2019) Current prophylactic measures and available vaccines have demonstrated limited efficacy in preventing the transmission and spread of *M. agalactiae.*(Corrales et al. 2007; Loria et al. 2019) Furthermore, CA often follows a cyclical pattern of remission and recrudescence, with asymptomatic windows during which infected animals can continue shedding mycoplasmas in milk at low bacterial loads.(Bergonier et al. 1997; Addis et al. 2011).

In this context, the development of reliable diagnostic tools is essential for the effective management of CA. The standard diagnostic approach relies on the isolation of the pathogen which, although cost-effective and technically accessible, is time-consuming and may require additional analyses for species confirmation. In recent years, several molecular and serological methods have been developed for both direct and indirect detection of *M. agalactiae*, including endpoint and real-time PCR assays as well as ELISA-based diagnostics.(Dedieu et al. 1995; Tola et al. 1996; Rosati et al. 2000; Greco et al. 2001; Fusco et al. 2007; Lorusso et al. 2007; Becker et al. 2012) However, widespread implementation of these methods at the individual animal level remains limited due to their relatively high cost and the large flock sizes, which can comprise up to 1,000 sheep. The use of bulk tank milk (BTM) could offer a practical and cost-effective strategy suitable for large-scale implementation of dairy herd health surveillance systems supporting CA control and eradication programs.(Nobrega et al. 2023; Rowe et al. 2024).

This study describes the development of a highly sensitive real-time PCR assay for the detection of *M. agalactiae* in BTM samples collected from sheep farms in Sardinia, enabling reliable identification of infection even in herds exhibiting low *M. agalactiae* prevalence. Following analytical validation of its specificity and sensitivity, the assay was employed in a large-scale epidemiological survey conducted on ovine farms in Sardinia. Implications for the development of regional surveys and for the implementation of broader BTM-based molecular surveillance systems at national and international levels are also discussed.

## Materials and methods

### Sample collection

Individual and bulk tank milk samples were randomly selected and provided by the Laore Laboratory (formerly ARAS, Associazione Regionale Allevatori della Sardegna, Regional Breeders Association of Sardinia), which conducts routine quality control testing. Samples were collected between spring 2014 and spring 2015 from 924 sheep farms (7.3% of all sheep farms in Sardinia). Moreover, BTM and 62 individual milk samples were obtained from a sheep farm located in Ottana (Nuoro, Sardinia).

### DNA extraction

Total DNA was extracted from 300 µL of individual or BTM with the MagMAX™ Express Magnetic Particle Processors (Applied Biosystems) using the dedicated kit and following vendor’s recommendations. DNA was then quantified with a NanoDrop Lite Spectrophotometer (Thermo Fisher) and stored at -20 °C until further analysis.

DNA from mycoplasmas was extracted with the DNeasy^®^ Blood & Tissue Kit (Qiagen) following vendor’s recommendations.

### Primers and probes design

Sequences of the *M. agalactiae p48* gene from the PG2^T^ type strain and different field isolates were retrieved from the GenBank (Supplementary file 1) and aligned using the MUSCLE online tool (https://www.ebi.ac.uk/jdispatcher/msa/muscle) (Madeira et al. 2024). Specific primers and the TaqMan™ MGB probe were designed on a conserved region of the *p48* gene by using the Primer Express Software v2.0 (Applied Biosystems). The *Ovis aries β-actin* gene (NC_056077.1:39220697–39224085) was used as housekeeping gene and primers and probe were designed with the same tool. Primers and probes sequences are reported in Table 1.

**Table 1:** Primers used in this work

Target gene	Name	Sequence (5’→3’)	Position	Amplicon size
p48	MAGP48/F	TTCAGGAACACCTCAAGCTACTACA	825–849	76 bp
p48	MAGP48/R	TGAACCAGCAACAGGGTAAGAA	879–900	76 bp
p48	MAGP48/Probe	6-FAM-TAACTCTGTGGTTAAAGCT	855–873	76 bp
β-actin	β-ACT/F	CGTCCGTGACATCAAGGAGAA	2054–2074	60 bp
β-actin	β-ACT/R	GCCATCTCCTGCTCGAAGTC	2094–2113	60 bp
β-actin	β-ACT/Probe	HEX- TCTGCTACGTGGCCC	2077–2091	60 bp

### Real-time PCR

Real-time PCR was conducted using the Luna^®^ Universal Probe qPCR Master Mix (New England Biolabs) according to the manufacturer’s instructions. Briefly, multiplex (*p48* + *β-actin*) or singleplex (only *p48*) reaction mixtures were prepared as follows: 10 µL of 2X Luna^®^ Universal Probe qPCR Mix, 0.4 µM each primer, 0.2 µM of each probe, 5 µL of template DNA (50 ng/µL), and nuclease-free water to reach the 20 µL final volume. The amplification was conducted in a Rotor-Gene Q instrument (Qiagen) using the following thermal cycling conditions: initial denaturation at 95 °C for 1 min followed by 40 cycles of 95 °C for 15 s and 60 °C for 30 s. Fluorescence was detected using green or yellow channel with gain set to 5 for *p48* and *β-actin* detection, respectively. Data analysis was performed using the Rotor-Gene Q Software version 2.3.1.49, setting the fluorescence threshold at 0.032 after the Noise Slope Correction.

All samples were tested in triplicate and appropriate positive, negative, and no template controls (NTC) were included in all experiments.

### p48 real-time PCR specificity and sensitivity

To evaluate specificity, genomic DNA was extracted from 3 *M. agalactiae* field isolates and from various other *Mycoplasma* species of the *M. hominis* group or infecting small ruminants (*M. spumans*, *M. arthritidis*, *M. bovis*, *M. capricolum*, *M. gypis*, *M. hominis*, *M. mycoides*, *M. salivarium*, and *M. conjunctivae*). Twenty ng replicates (3) of genomic DNA were tested by *p48* real-time PCR. Positive amplicons were Sanger-sequenced. Additionally, an in silico BLAST analysis of the of the newly designed primers against the entire NCBI database was conducted.

The pVAX1/*p48* plasmid (Chessa et al. 2009) was used to generate a quantitative standard curve. Serial 10-fold dilutions ranging from 1 × 10⁷ copies to a single copy of the target sequence were prepared in TE buffer to generate a standard curve. Dilutions were analysed in triplicate by real-time PCR and the limit of detection (LOD) was determined. To evaluate the performance of the assay throughout the entire workflow, the same plasmid dilutions were spiked into 300 µL aliquots of pasteurized milk. DNA was extracted as described above and then subjected to real-time PCR under the same conditions used for the standard curve. For both curves, slope and PCR efficiency was calculated.

The sensitivity of the method was also assessed in relation to within-herd *M. agalactiae* prevalence, with the aim of determining the minimum number of infected sheep contributing to BTM required for a positive BTM test result. Briefly, BTM and 62 individual milk samples coming from the Ottana herd were tested by real-time PCR and classified as negative (not determined, ND), positive (Ct ≤ 35), or borderline (Ct > 35). The prevalence of *M. agalactiae* was calculated. Three different simulated BTM samples (namely M1, M2, and M3) were prepared by mixing positive and negative samples in order to reach the real prevalence in the herd, previously calculated by testing the 63 individual samples. The same negative samples were used for all 3 mixes, while the positive samples were chosen randomly for each mix. Simulated and real BTM samples were diluted to 1:5, 1:10, 1:25, 1:50 in *M. agalactiae*-negative milk. All these samples were subjected to DNA extraction and *p48* real-time PCR, as previously described. All samples were tested in triplicate. Graphics were generated with the GraphPad Prism 5.04 software.

### Epidemiological investigation

DNA samples obtained from 924 BTM samples collected from 924 sheep farms in Sardinia, were tested by *M. agalactiae p48*-based real-time PCR. Samples were classified as negative (ND), positive (Ct ≤ 35), or borderline (Ct > 35). The online tool Map Maker (https://maps.co/gis/) was used to to illustrate the geographical coverage of the survey and the distribution of positive detections and generate the GIS map.

## Results

### Specificity and sensitivity assessment

The specificity of the *p48* real-time PCR assay was evaluated by testing DNA from multiple non-target *Mycoplasma* species together with 3 *M. agalactiae* field isolates. *M. spumans*,* M. arthritidis*,* M. bovis*,* M. capricolum*,* M. gypis*,* M. hominis*,* M. mycoides*,* M. salivarium*, and *M. conjunctivae* were always PCR negative, while all *M. agalactiae* field isolates generated a positive signal in real-time PCR (data not shown). Moreover, the specificity of the amplicon was confirmed by Sanger sequencing. No amplification was observed in NTCs, confirming the absence of artifacts or primers dimers. Moreover, the in silico BLAST analysis confirmed that the *p48* primers have both 100% identity and coverage only with *M. agalactiae* strains.

**Fig. 1:**
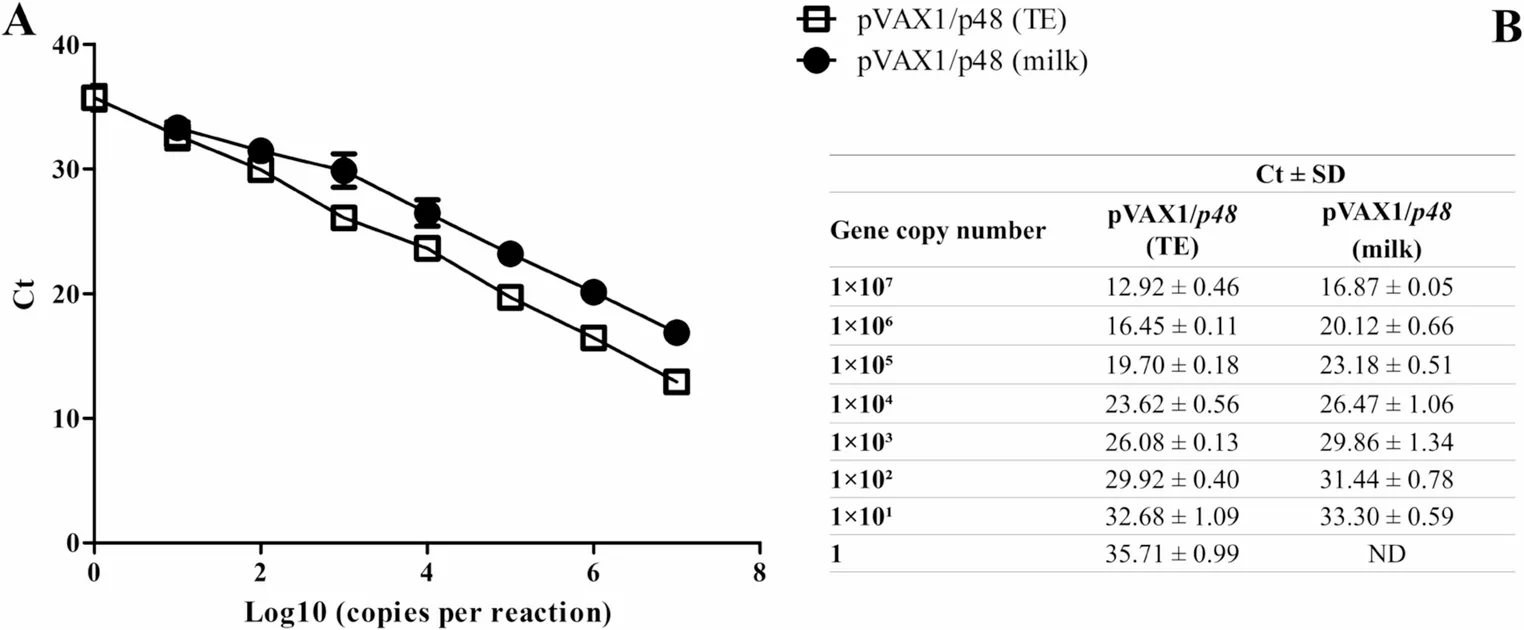
Sensitivity of the *M. agalactiae* p48-based real-time PCR. (**A**) Standard curves of pVAX1/p48 diluted in TE buffer (empty squares) or milk (black circles). (**B**) Mean Ct values (3 replicates) of pVAX1/p48 serial dilutions in TE or in milk matrix. Data are expressed as Ct values ± SD

To evaluate *p48* real-time PCR sensitivity, 10-fold serial dilutions (1 × 10⁷ to 1 copy per reaction) of the pVAX1/*p48* plasmid were prepared both in TE buffer and in milk. Standard curves were generated and linearity was observed across the dilution series (ranging from 1 × 10^0^ to 1 × 10^7^) in both matrices (Fig. 1), with R² values of 0.998 and 0.986 for the TE buffer and milk preparations, respectively. Ct values were consistently higher in milk than in TE buffer. Samples were considered positive with Ct ≤ 35 while samples with 35 < Ct ≤ 40 were classified as borderline. Slope and PCR efficiency were calculated for both standard curves; the TE dilutions curve had a slope of -3.2876 and a PCR efficiency of 101.3%, while the milk dilutions curve showed a -2.785 slope and a 129% PCR efficiency. The milk dilutions standard curve was generated again considering only the 1 × 10^1^ to 1 × 10^7^ range, generating a curve with a -3.269 slope and a 101.6% PCR efficiency (Supplementary material).

The limit of detection (LOD) was determined at 10 copies per reaction in both matrices. At this concentration, the assay yielded mean Ct values of 32.68 (± 1.09 SD) in TE buffer and 33.30 (± 0.59 SD) in milk, with low coefficients of variation (3.3% and 1.8%, respectively).

**Fig. 2:**
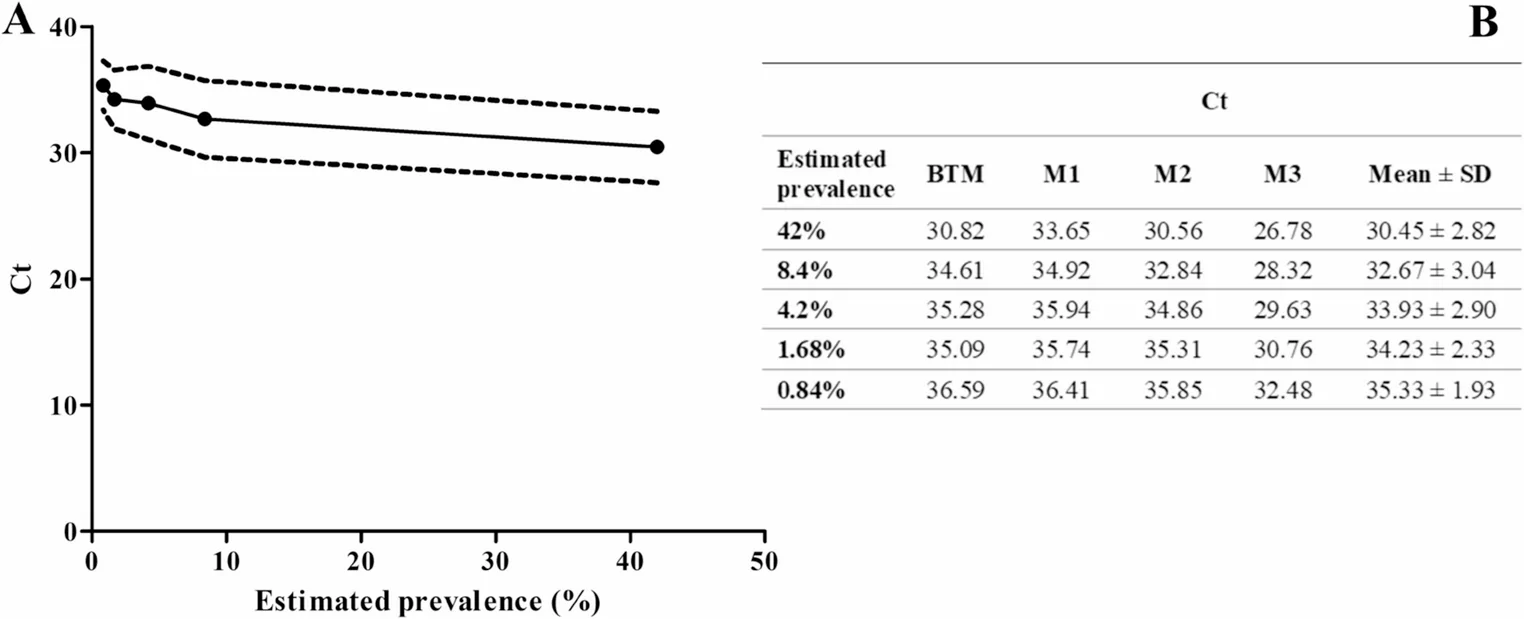
Relationship between estimated within-flock prevalence and Ct values obtained from real and simulated BTM samples. (**A**) Association between the estimated prevalence of *M. agalactiae*-positive animals within a flock and the corresponding mean Ct values obtained for real and simulated BTM dilutions at different estimated prevalence (%). Dashed lines represent the SD. (**B**) Mean Ct values obtained by real-time PCR for serial dilutions of the real BTM and the 3 simulated mixes (M1, M2, and M3)

The minimum number of infected sheep contributing to BTM required for a positive BTM test result was evaluated. First, the *M. agalactiae* prevalence was determined within the Ottana herd by testing 62 individual milk samples. Real-time PCR identified 26 (41.93%) positive, 3 (4.84%) borderline, and 33 (53.23%) negative samples, establishing *M. agalactiae* prevalence in this flock at approximately 42%. All samples were positive for the β-actin endogenous control, with Ct values ranging from 23 to 26, confirming the integrity of the extracted nucleic acids. While β-actin amplification confirmed the presence of amplifiable ovine DNA, this endogenous control does not monitor extraction efficiency or inhibition. For definitive diagnostic use, an exogenous spike-in control is recommended. Starting from these data, 3 mixes (namely M1, M2, and M3) were created simulating 3 BTM samples with 42% prevalence. Dilutions of the BTM and M1, M2, and M3 were obtained and tested by real-time PCR (see Materials and Methods). Figure 2 shows the relationship between estimated prevalence (varying from 42% to 0.84%) and Ct values obtained from dilutions of both simulated and real BTM samples. Similar trends were observed across all samples, with BTM, M1, and M2 showing comparable Ct values across the considered prevalence levels. In contrast, M3 consistently exhibited lower Ct values, suggesting a higher concentration of *M. agalactiae*. The minimum detectable infected-animal contribution under the experimental conditions tested was established at 1.68% prevalence, even if an amplification signal was constantly detected also with simulated prevalence < 1%.

### Epidemiological investigation

**Fig. 3:**
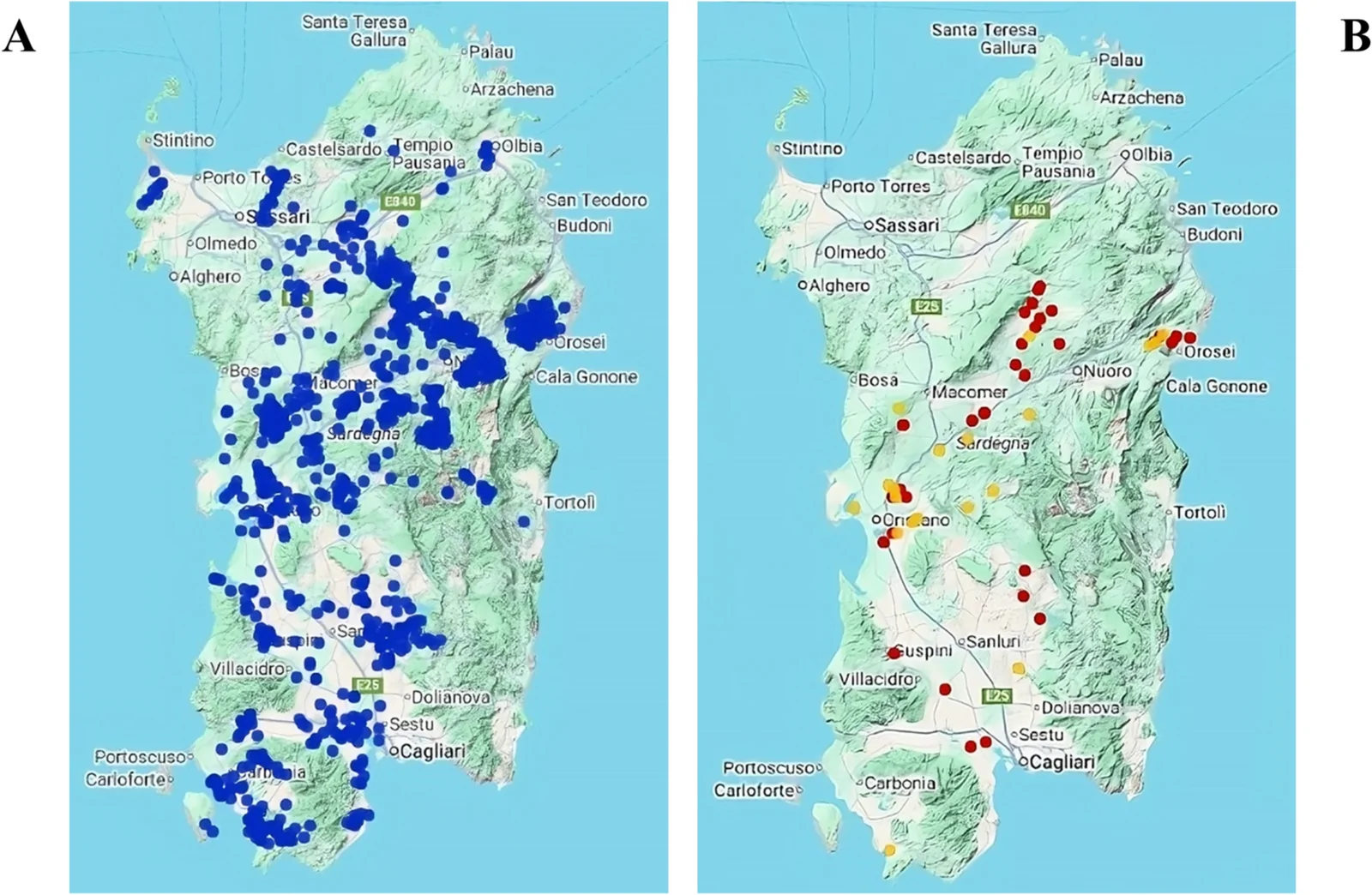
Geographical distribution of sheep dairy farms included in the screening across Sardinia. Blue dots indicate screened farms; positive (red) and borderline (yellow) farms are reported

The newly developed real-time PCR assay was used to perform an epidemiological survey on *M. agalactiae* in Sardinia. We screened 924/12,560 (7.3%) ovine flocks, located across 156 municipalities. Among the tested flocks, 34 (3.68%) tested positive for *M. agalactiae*, 19 (2.06%) yielded borderline results, and 871 (94.26%) tested negative. The geographical distribution of the total sampled farms and the positive and borderline flocks is shown in Fig. 3. Data confirmed that *M. agalactiae* is geographically widespread in Sardinia. The herd-level prevalence was estimated at 3.68% when only strongly positive samples were considered; when weakly positive (borderline samples) were also included, the estimated prevalence increased to 5.73%.

## Discussion

Contagious agalactia (CA) remains one of the most significant infectious diseases affecting small ruminant dairy systems worldwide, particularly in endemic regions of the Mediterranean area. The chronic and recurrent nature of *M. agalactiae* infections, coupled with its ability to persist and be shed intermittently in milk, pose major challenges for disease control and eradication. Traditional bacteriological culture, although specific, is laborious and time-consuming, and its diagnostic sensitivity is often limited by intermittent bacterial shedding and overgrowth of contaminants.(Bergonier et al. 1997; Loria et al. 2019) Molecular techniques, and especially real-time PCR, have emerged as powerful tools for the rapid and specific detection of *M. agalactiae*,(Lorusso et al. 2007; Becker et al. 2012) yet their widespread application at the individual-animal level remains limited due to the high cost in large flocks.(Nobrega et al. 2023).

The present study aimed to overcome these constraints by developing and validating a novel *p48*-based real-time PCR assay for the detection of *M. agalactiae* in bulk tank milk (BTM). The assay demonstrated high analytical specificity, as no amplification was observed in DNA from phylogenetically related *Mycoplasma* species nor was predicted by in silico BLAST analysis. The absence of false-positive signals or primers dimer artifacts confirmed the robustness of the assay design. The assay exhibited high analytical sensitivity, with a limit of detection (LOD) of 10 copies per reaction in both TE buffer and milk matrices and a linear dynamic range of 1 × 10^0^-1 × 10^7^ and 1 × 10^2^ to 1 × 10^7^, respectively. Although a linear relationship was observed across these ranges, the assay was designed for qualitative rather than quantitative detection. The PCR efficiency was in the acceptable range for both curves, according to MIQE guidelines.(Bustin et al. 2009) The slightly higher Ct values observed in milk compared with TE buffer likely reflect moderate DNA loss during extraction.

At the herd level, the assay successfully detected *M. agalactiae* DNA in both simulated and real BTM samples, even at a within-herd prevalence as low as 1.68%. This result is highly relevant from a diagnostic perspective, since *M. agalactiae* prevalence in lactating ewes during CA outbreaks can range from 1% to 50%.(Bergonier et al. 1997) The consistent amplification profiles obtained from simulated and real BTM samples indicate a high degree of assay reproducibility and robustness under field conditions, where heterogeneity in milk yield and pathogen shedding is expected.(Addis et al. 2011) This high sensitivity suggests that the assay could serve as an early-warning tool for herd-level screening, detecting infection even in subclinical or pre-epidemic phases.

The large-scale application of this diagnostic method in Sardinia provided valuable epidemiological insights. The detection of *M. agalactiae* in 3.68% of flocks based on strongly positive BTM samples confirms that the pathogen remains endemic in the region, albeit at relatively low levels outside outbreak periods. Inclusion of weakly positive (borderline) samples increased the estimated herd-level prevalence to 5.73%, without substantially altering the interpretation of the epidemiological status of the region.These findings are consistent with earlier reports of persistent, low-level circulation of *M. agalactiae* in Mediterranean small ruminants.(De la Fe et al. 2005; Ariza-Miguel et al. 2012; Migliore et al. 2021) It should be noticed that to date the epidemiological situation can be different, but the presented data represent the first large scale implementation of a *M. agalactiae* assay based on BTM. Sardinia’s dense dairy sheep population, uniform management systems, and well-established milk collection network make it an ideal setting for implementing BTM-based molecular surveillance.

Importantly, BTM testing represents a cost-effective and logistically feasible approach for large-scale monitoring of CA. Compared with individual milk testing, BTM analysis integrates milk from the entire herd, providing a representative assessment of infection status while significantly reducing costs.(Rowe et al. 2024) This strategy can be readily integrated into existing milk quality and somatic cell count monitoring programs, enhancing early detection capacity and supporting regional control efforts. The adoption of molecular BTM testing could thus strengthen CA surveillance programs, both in endemic regions such as Sardinia and in other European and Mediterranean countries where CA remains a notifiable disease.(Bergonier et al. 1997) However, shedding intensity varies substantially among infected sheep and BTM results should be interpreted as semi-quantitative indicators of herd infection status rather than precise prevalence estimates.

In conclusion, the *p48* real-time PCR assay described here exhibits high specificity, sensitivity, and reproducibility, making it a valuable tool for the detection of *M. agalactiae* in BTM. Its demonstrated capacity to identify infection at low prevalence highlights its utility for early detection, herd-level surveillance, and control of CA. Integration of this assay into existing milk monitoring frameworks could substantially improve diagnostic efficiency and contribute to the long-term management and reduction of CA in small ruminant populations.

## Conclusions

The p48-based real-time PCR assay developed in this study provides a reliable method for the detection of *M. agalactiae* in bulk tank milk, combining high analytical performance with suitability for large-scale herd screening. Its ability to detect infection in pooled milk samples supports the use of BTM as a practical matrix for monitoring contagious agalactia at the herd level.

Application of the assay to a large Sardinian flock population confirmed the continued circulation of *M. agalactiae* and demonstrated the feasibility of implementing molecular BTM surveillance under field conditions. By enabling efficient and cost-effective screening of dairy sheep flocks, this approach may facilitate earlier identification of infected herds and support evidence-based disease control programs.

Overall, the study highlights the potential of BTM-based molecular surveillance as a valuable component of contagious agalactia monitoring strategies in endemic regions and provides a framework for its wider adoption in small-ruminant production systems. 

## Supplementary Information

Below is the link to the electronic supplementary material.

Supplementary Material 1 (TXT 40.3 KB)

Supplementary Material 2 (PNG 1.23 MB)

High Resolution Image (TIF 391 KB)

## Data Availability

No datasets were generated or analysed during the current study.
